# Assessment of salivary matrix metalloproteinase (MMP8) and activated salivary matrix metalloproteinase (aMMP8) in periodontitis patients: a systematic review and meta-analysis

**DOI:** 10.3389/froh.2025.1444399

**Published:** 2025-02-19

**Authors:** Sean G. Boynes, Nigar Sofiyeva, Tina Saw, Valerie Nieto, Leena Palomo

**Affiliations:** ^1^ GameShift Healthcare Solutions, LLC, Harmony Health, Inc., Weirton, WV, United States; ^2^ Department of Clinical Science, University of Bergen, Bergen, Norway; ^3^ Oral Genome, Harmony Health, Inc., Carlsbad, CA, United States; ^4^ Dental Hygiene, University of Michigan, American Institute of Dental Public Health, Ann Arbor, MI, United States; ^5^ Department of Periodontology & Implant Dentistry, NYU College of Dentistry, New York, NY, United States

**Keywords:** salivary diagnostics, periodontitis, biomarkers, matrix metalloproteinase 8 (MMP-8), metaanalysis, saliva, disease progression, oral health

## Abstract

**Introduction:**

Periodontitis affects a significant portion of the global population and is associated with systemic health issues. Salivary biomarkers such as salivary matrix metalloproteinase-8 (MMP-8) and its activated form (aMMP-8) have been studied for their roles in tissue degradation and inflammation in periodontitis. This meta-analysis investigates the association between salivary MMP-8 and aMMP-8 levels and periodontitis.

**Methods:**

A systematic literature search was conducted utilizing PubMed, Embase, Web of Science, and Cochrane Library databases up to October 2023, yielding 35 studies that quantified MMP-8 or aMMP-8 in saliva from patients with periodontitis and healthy controls. Data were extracted, and standardized mean differences (SMD) with 95% confidence intervals (CI) were calculated. Heterogeneity was assessed, and subgroup analyses were performed based on saliva collection techniques. Meta-regression analysis evaluated the impact of publication year on heterogeneity.

**Results:**

The meta-analysis included 35 studies. Pooled results indicated significantly higher levels of MMP-8 and aMMP-8 in periodontitis cases compared to healthy controls (SMD: 2.71, 95% CI: 1.04–4.38, *p* = 0.002) with substantial heterogeneity (I^2^ = 94.5%). No significant difference was found between MMP-8 and aMMP-8 (*p* = 0.445). Subgroup analyses by saliva collection technique did not reduce heterogeneity significantly. Meta-regression showed that publication year did not impact heterogeneity. Small-study effects and publication bias were present, suggesting caution in interpreting the results.

**Discussion:**

The findings support the potential of MMP-8 and aMMP-8 as biomarkers for periodontitis, although substantial heterogeneity and methodological differences among studies pose challenges. Standardized protocols and larger sample sizes are necessary to enhance the reliability of these biomarkers in clinical practice. Despite limitations, salivary diagnostics hold promise for non-invasive, early detection and monitoring of periodontitis.

**Conclusion:**

Salivary MMP-8 and aMMP-8 levels are significantly associated with periodontitis, highlighting their potential as diagnostic biomarkers. However, methodological improvements and standardization are essential for their clinical application. Collaborative efforts and advancements in salivary diagnostics are crucial for improving periodontitis management and patient outcomes.

## Introduction

Periodontitis stands as a significant public health concern, effecting an estimated 20%–50% of the global population (1, 2). This inflammatory disease affects systemic health beyond the oral cavity, highlighting the importance of early diagnosis (3, 4). Key to its pathogenesis are molecular mechanisms involving salivary matrix metalloproteinase-8 (MMP-8) and its activated form (aMMP-8), which contribute to tissue degradation and inflammation (5).

Salivary matrix metalloproteinases (MMPs) constitute a family of zinc-dependent endopeptidases crucial for extracellular matrix remodeling and turnover. Among them, MMP-8, also known as neutrophil collagenase, plays a pivotal role in the degradation of collagen types I, II, and III, key components of periodontal tissues (6, 7). It is predominantly produced by neutrophils, but other cell types, including fibroblasts and macrophages, also contribute to its expression. Activated MMP-8 (aMMP-8) arises from the cleavage of pro-MMP-8 by other proteases, such as serine proteases or other MMPs. This activation process unleashes the proteolytic potential of MMP-8, enhancing its ability to degrade extracellular matrix components (8). The presence of aMMP-8 in periodontal tissues underscores its role as a key mediator of tissue destruction in periodontitis (9, 10).

Periodontitis, characterized by inflammation and destruction of periodontal tissues, represents a complex interplay between microbial pathogens and host immune response. MMP-8 and aMMP-8, with their capacity to degrade collagen and other extracellular matrix components, are implicated in the breakdown of periodontal tissues, leading to clinical manifestations such as gingival inflammation, periodontal pocket formation, irreversible bone loss, and, ultimately, tooth loss. Therefore, the earliest diagnosis will result in better outcomes for an individual at risk for the progression of periodontitis. While clinical and radiological criteria aid in diagnosing periodontitis, detecting it early and tracking its progression can be difficult. MMP-8 shows promise as the top biomarker for predicting, diagnosing, and gauging the progression of periodontitis (11–13).

Saliva has emerged as a pivotal diagnostic medium in dentistry due to its non-invasive collection method and the presence of a plethora of biomarkers. Saliva collection methods are generally categorized as stimulated or unstimulated and have the capability to conduct point-of-care (chairside) testing (14–17). Furthermore, its utility extends beyond traditional clinical assessments, offering insights into various oral and systemic diseases, including periodontits (18). Recent advancements in technologies, such as saliva-based diagnostics approaches, have propelled saliva to the forefront of dental research (19). These methodologies enable the identification of microbial signatures and the quantification of specific protein biomarkers like MMP-8 and aMMP-8, elucidating disease pathogenesis and progression (20, 21). The integration of saliva-based diagnostics into clinical practice holds promise for early disease detection, personalized treatment strategies, and improved patient outcomes (22, 23). It should be noted, to date, there are no FDA approved testing methods or devices for the evaluation of periodontitis risk. The current marketplace features methods and devices categorized within the FDA's Health and Wellness device category. This category correlates to low-risk products that promote a healthy lifestyle (general wellness products) (24).

In our systematic meta-analysis, we investigated the potential association between salivary MMP-8 and aMMP-8 levels and periodontitis by examining a total of 35 relevant studies. Our analysis aimed to distinguish any discernible patterns or trends in MMP-8 and aMMP-8 levels among individuals with periodontitis compared to those without. Additionally, we explored the utility of saliva as a non-invasive diagnostic medium for periodontitis. The findings from this study provide valuable insights into the potential role of MMP-8 as a biomarker and the broader implications of salivary analysis in periodontitis diagnosis.

## Materials and methods

We conducted a systematic literature search and meta-analysis to investigate the role of salivary matrix metalloproteinase (MMP8) and activated matrix metalloproteinase (aMMP8) in periodontitis according to Preferred Reporting Items for Systematic Reviews and Meta-Analyses (PRISMA 2020) Statement (25). Medical databases, including PubMed, Embase, Web of Science, and Cochrane Library, were searched from database inception until October 2023. The comprehensive list of search strategies is presented in Appendix 1. Literature was imported to the EndNote X9 referencing program (The EndNote Team, Clarivate, Philadelphia, PA, USA), and literature was deduplicated using a “Find Duplicates” function. Unique studies were screened based on the inclusion criteria. Original data reporting MMP8 and aMMP8 levels measured in the saliva of patients diagnosed with periodontitis and healthy controls were eligible for the analysis.

Studies excluded during screening included case reports, reviews, non-English publications, unpublished data, those using gingival fluid or serum for MMP8/aMMP8 analysis or mRNA and protein expression data. The remaining studies were screened with full-text information, and extracted data was used in data synthesis. In case of data presented from the same center and overlapping time frame, the latest or the largest dataset was included in the analysis.

Due to the different unit and measurement methods across eligible studies, the effect size was reported in standardized mean differences (SMD) and 95% confidence intervals (CI) (26). Mean and standard deviations were calculated using Hozo's equation if the data were presented in median, minimum, and maximum values (27). Data were extracted from bar plots using the online tool available at WebPlotDigitizer - Copyright 2010–2023 Ankit Rohatgi (automeris.io), when needed. Studies reporting medians with interquartile ranges (IQR) were excluded due to the precautions in converting IQR to standard deviation according to the Cochrane Handbook for Systematic Reviews of Interventions (version 6.4, 2023) (28). The significance level was set to an alpha value of 0.05. The heterogeneity between studies was measured with *I*^2^ values.

The PRISMA flowchart was generated using a Shiny app by Haddaway et al. (29). Statistical analysis and data visualization were performed using RStudio 2023.12.1 (R version 4.3.1) statistical software (R Core Team, Vienna, Austria) (30). Meta-analysis was performed using the *meta* package (31, 32). The outlier studies were determined using the *dmetar* package (33, 34). Results of meta-analysis and small study effects were illustrated with Forest and contour-filled Funnel plots, respectively. The Funnel plot asymmetry was analyzed using Egger's asymmetry test (35–38), and the small-study effect was adjusted with Duval and Tweedie trim and fill method (39, 40).

## Results

**Figure 1:**
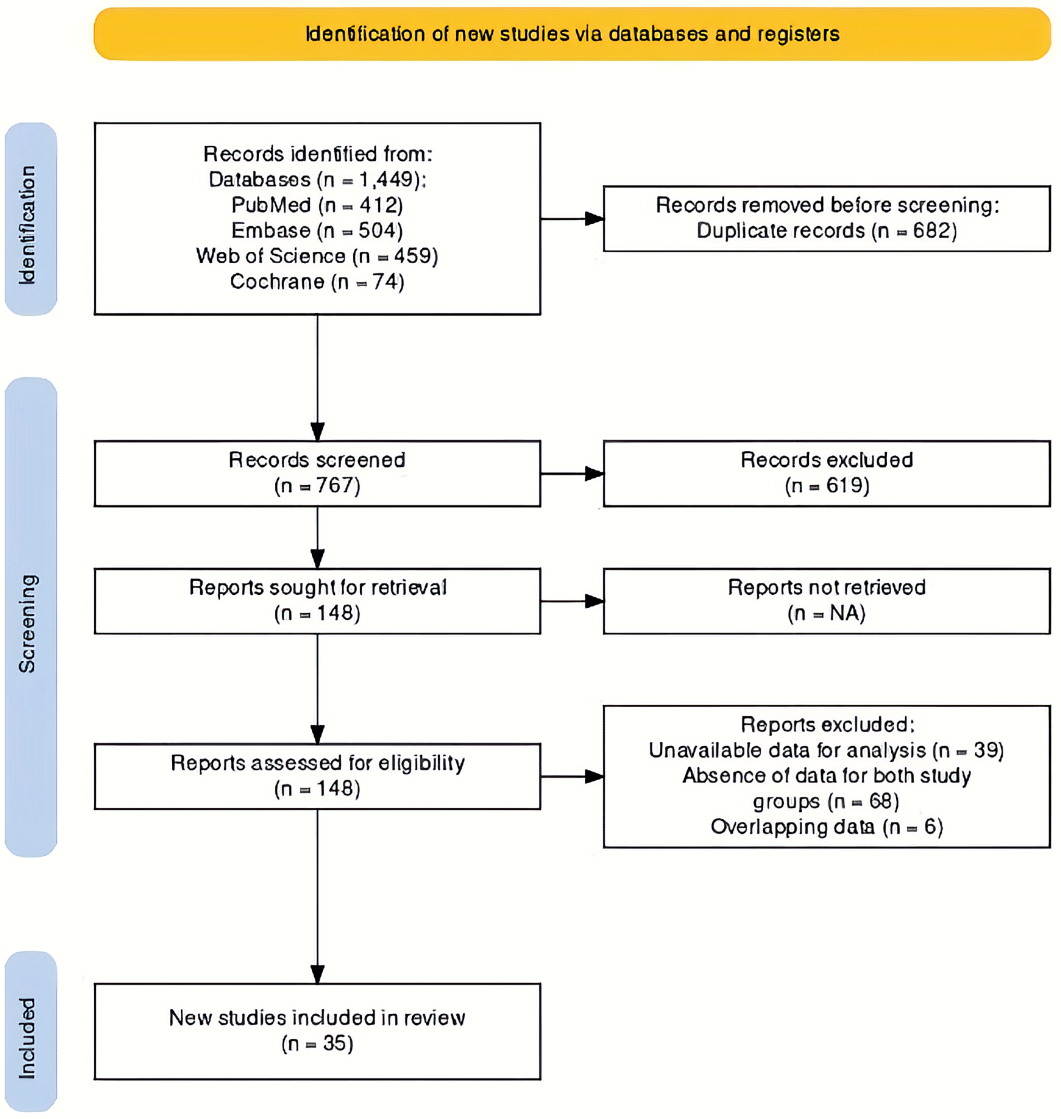
PRISMA flowchart illustrating the literature screening process.

Systematic literature screening of 767 unique studies yielded 148 studies for full-text screening. The largest group of excluded studies was the ones reporting data from periodontitis patients or healthy controls only (*n* = 68). The following most common reason was the unavailability of reported data required for statistical analysis (*n* = 39). The PRISMA Flowchart illustrating the screening process is shown in Figure 1.

We found 25 eligible studies published between 2008 and 2022 reporting MMP8 values in periodontitis cases (*n* = 1317) with matched healthy controls (*n* = 1,047) (41–65). MMP8 was measured in unstimulated saliva in 68% of included studies (*n* = 17), and ELISA was the most used method for quantification (Table 1).

**Table 1:** Studies included in the meta-analysis analyzing MMP8 and aMMP8 levels in patients with periodontitis and healthy controls.

Author, reference nr.	Publication year	Country	Measured parameter	Saliva sampling method	Method of measurement	Unit of measurement
Rai et al. (41)	2008	India	MMP8	Unstimulated	ELISA	ng/ml
Mirrielees et al. (42)	2010	USA	MMP8	Unstimulated	ELISA	ng/ml
Buduneli et al. (43)	2011	Türkiye	MMP8	Stimulated	IFMA	ng/ml
Rathnayake et al. (46)	2013	Sweden	MMP8	Stimulated	ELISA	ng/ml
Ebersole et al. (44)	2013	USA	MMP8	Unstimulated	ELISA	ng/ml
Meschiari et al. (45)	2013	Brazil	MMP8	Stimulated	ELISA	ng/ml
Miricescu et al. (47)	2014	Romania	MMP8	Unstimulated	ELISA	ng/mg albumin
Gupta et al. (50)	2015	India	MMP8	Unstimulated	ELISA	ng/ml
Ebersole et al. (49)	2015	USA	MMP8	Unstimulated	Millipore	ng/ml
Akbari et al. (48)	2015	India	MMP8	Unstimulated	ELISA	ng/ml
Martinez et al. (52)	2017	Sweden	MMP8	Unstimulated	ELISA	ng/ml
Lira-Junior et al. (51)	2017	Türkiye	MMP8	Unstimulated	ELISA	ng/ml
Rangbulla et al. (53)	2017	India	MMP8	Unstimulated	ELISA	ng/ml
Golitsyna et al. (54)	2018	Russia	MMP8	Not reported	ELISA	ng/ml
Sharma et al. (55)	2018	India	MMP8	Unstimulated	ELISA	ng/*μ*l
Aungurencei et al. (56)	2019	Romania	MMP8	Not reported	ELISA	ng/ml
Fastovets et al. (57)	2020	Ukraine	MMP8	Not reported	ELISA	mkg/L
Fatemi et al. (58)	2020	Iran	MMP8	Unstimulated	ELISA	ng/ml
Lee et al. (59)	2020	South Korea	MMP8	Unstimulated	ELISA	ng/ml
Lira-Junior et al. (62)	2021	United Kingdom	MMP8	Unstimulated	ELISA	ng/ml
Bostanci et al. (61)	2021	Türkiye	MMP8	Unstimulated	ELISA	ng/ml
Al Jasser et al. (60)	2021	Saudi Arabia	MMP8	Unstimulated	ELISA	ng/ml
Zhang et al. (63)	2021	China	MMP8	Stimulated	ELISA	ng/ml
Balaji et al. (64)	2022	India	MMP8	Unstimulated	ELISA	pg/ml
Wu et al. (65)	2022	China	MMP8	Not reported	ELISA	NA
Noack et al. (66)	2017	Germany	aMMP8	Both	ELISA	ng/ml
Raisanen et al. (15)	2019	Finland	aMMP8	Not Reported	IFMA	ng/ml
Heikkinen et al. (67)	2019	Finland	aMMP8	Stimulated	Not Reported	μg/L
Sorsa et al. (10)	2020	Finland	aMMP8	Not Reported	IFMA	ng/ml
Yucel et al. (68)	2020	Türkiye	aMMP8	Unstimulated	IFMA	ng/ml
Ozturk et al. (69)	2021	Türkiye	aMMP8	Not Reported	IFMA	ng/μl
Ramenzoni et al. (70)	2021	Switzerland	aMMP8	Both	ELISA	ng/ml
Deng et al. (71)	2022	China	aMMP8	Unstimulated	Lateral flow immunoassay	ng/ml
Umeizudike et al. (21)	2022	United Kingdom	aMMP8	Unstimulated	IFMA	ng/ml
Yilmaz et al. (72)	2023	Türkiye	aMMP8	Unstimulated	IFMA	ng/ml

 As the second outcome of interest, we analyzed activated MMP8 (aMMP-8) levels in periodontitis patients (*n* = 573) and healthy participants (*n* = 364). The literature review resulted in the inclusion of ten eligible studies reporting aMMP8 levels published between 2017 and 2023. (10, 15, 21, 66–72). Two of these studies reported data from two separate cohorts with stimulated and unstimulated saliva collection (Table 1).

**Figure 2:**
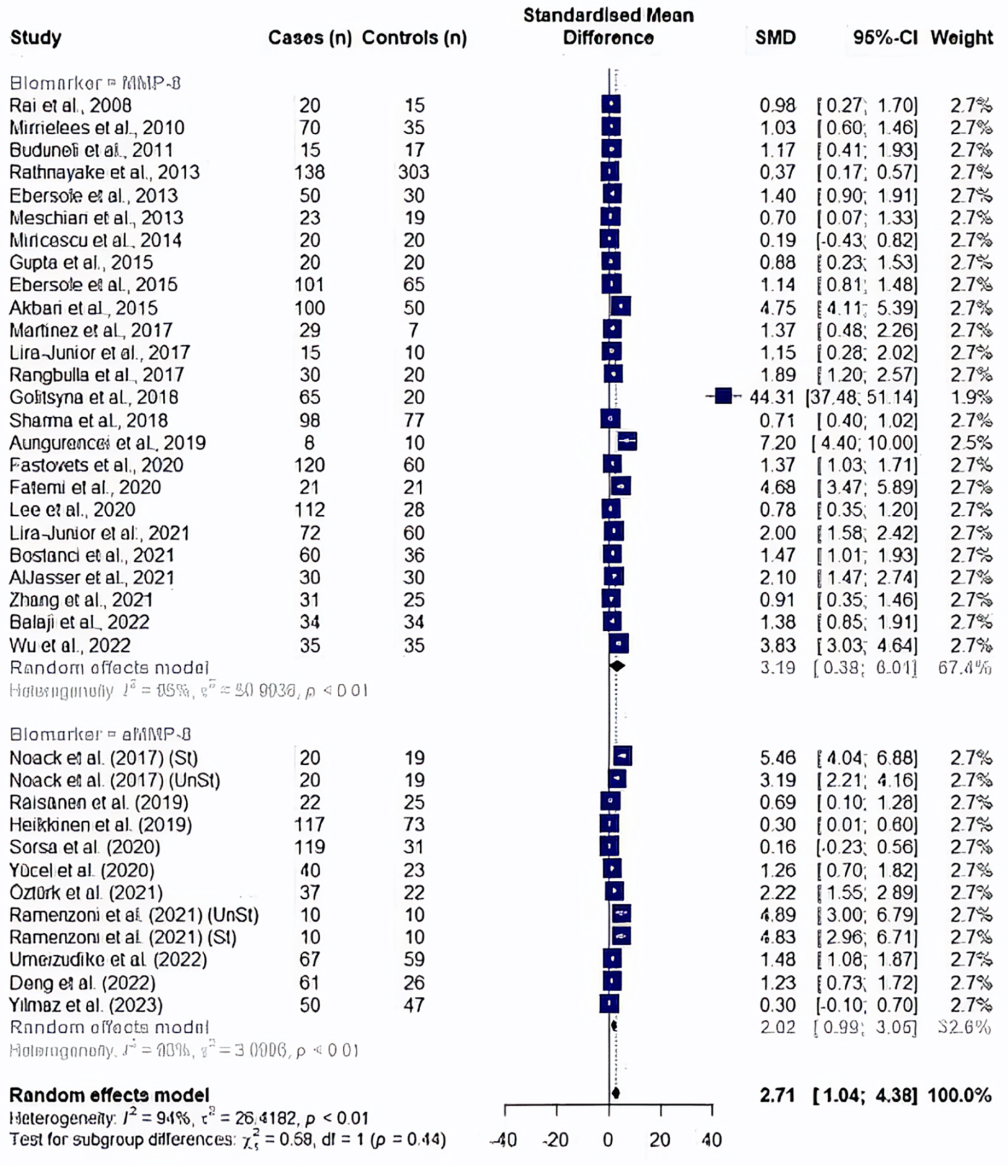
Forest plot illustrating a meta-analysis of MMP8 and aMMP8 values in saliva samples of periodontitis patients and healthy controls. St, stimulated saliva collection; UnSt, unstimulated saliva collection.

A random effect model meta-analysis of 35 studies with 37 study cohorts quantifying both biomarkers showed significantly high values in periodontitis patients with an overall standardized mean difference of 2.71 (95% CI: 1.04–4.38, *p* = 0.002), although with a high heterogeneity (*I*^2^ = 94.5%) (Figure 2). In a subgroup analysis by the type of the biomarker, studies evaluating MMP8 values showed an overall standardized mean difference of 3.19 (95% CI: 0.38–6.01, *p* = 0.026, *I*^2^ = 95%), while the effect size for aMMP8-quantifying cohorts was 2.02 (95% CI: 0.99–3.05, *p* < 0.001, *I*^2^ = 93.1%) (Figure 2). Overall, no statistical difference was found between studies measuring MMP8 and aMMP8 as a salivary biomarker (*p* = 0.445).

**Figure 3:**
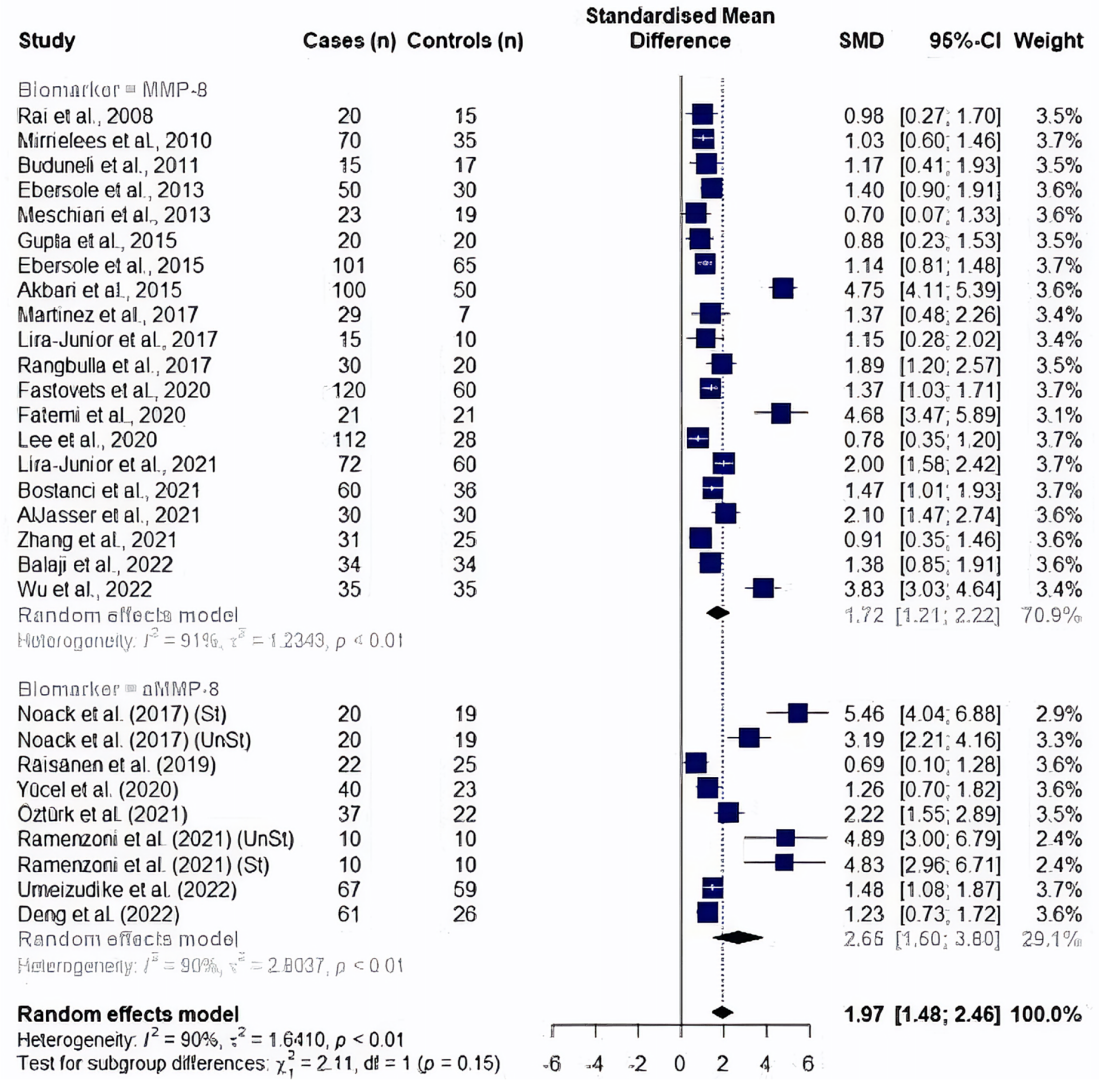
Forest plot illustrating a meta-analysis of MMP8 and aMMP8 values in saliva samples of periodontitis patients and healthy controls after removing outlier studies. *St, stimulated saliva collection; UnSt, unstimulated saliva collection*.

In a *post hoc* analysis, a study by Golitsyna et al. (54) showed an extremely large effect by exceeding the upper bound of the pooled effect and was accepted as an outlier study according to the definition by Viechtbauer et al. (73). An outlier finding function of *dmetar* package has confirmed the same study as an outlier affecting the random effect analysis results, in addition to seven other studies (10, 46, 47, 55, 56, 67, 72). After the removal of outlier studies (*n* = 29), a random effect meta-analysis yielded a significantly high MMP8 and aMMP8 levels in periodontitis patients (SMD: 1.97, 95% CI: 1.48–2.46, *p* < 0.0001, *I*^2^ = 90.2%). A subgroup of cohorts showed no difference between two salivary biomarkers (*p* = 0.147); MMP8: SMD = 1.72, 95% CI: 1.21–2.22, *p* < 0.0001, *I*^2^ = 90.8%) and aMMP8: SMD = 2.65, 95% CI 1.50–3.80, *p* < 0.0001, *I*^2^ = 90%) (Figure 3).

**Figure 4:**
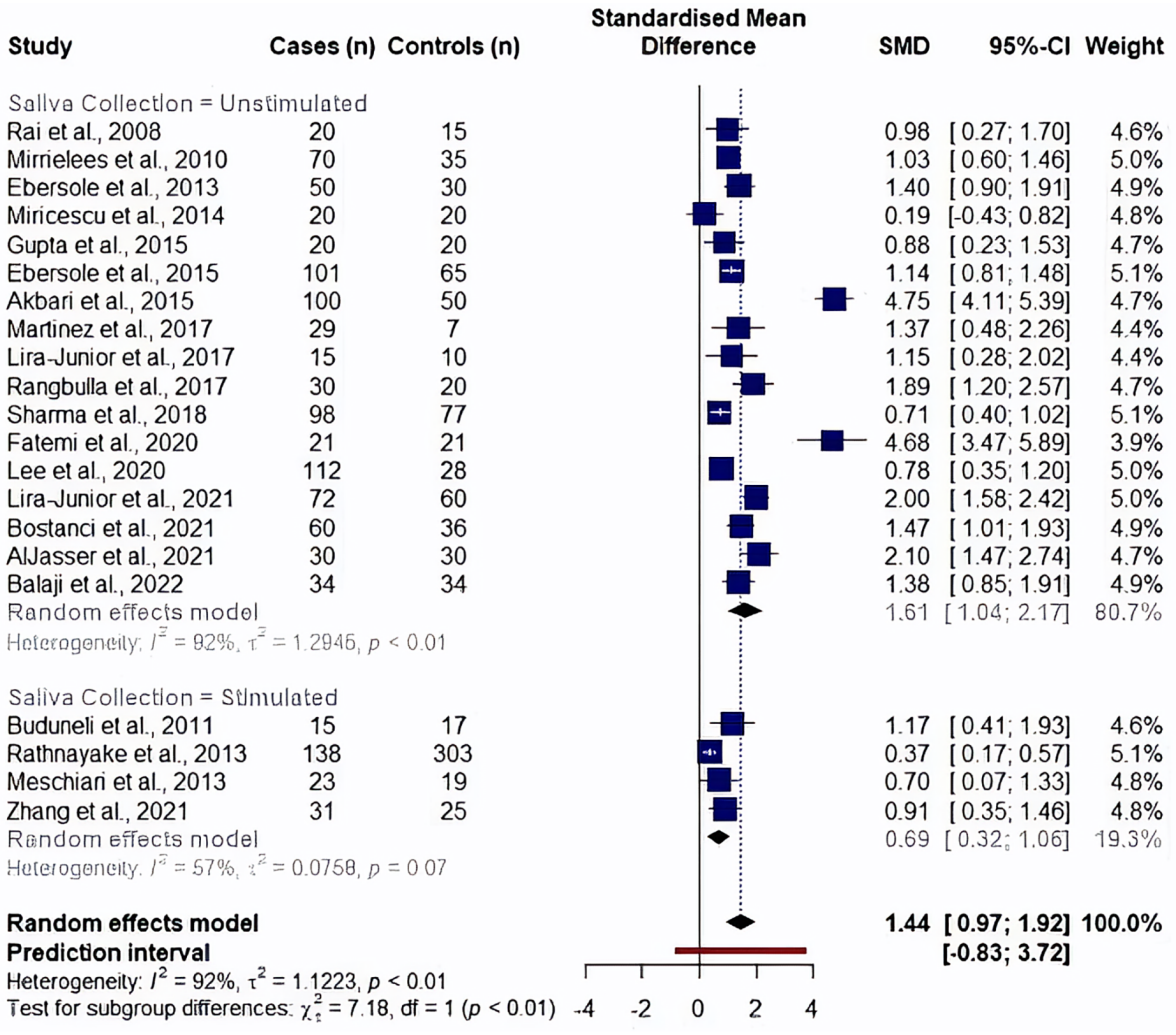
Subgroup analysis by saliva collection method evaluating MMP8 levels. Information on saliva collection method was not reported in studies by Golitsyna et al., 2018 (36), Aungurencei et al., 2019 (38), Fastovets et al., 2020 (39).

**Figure 5:**
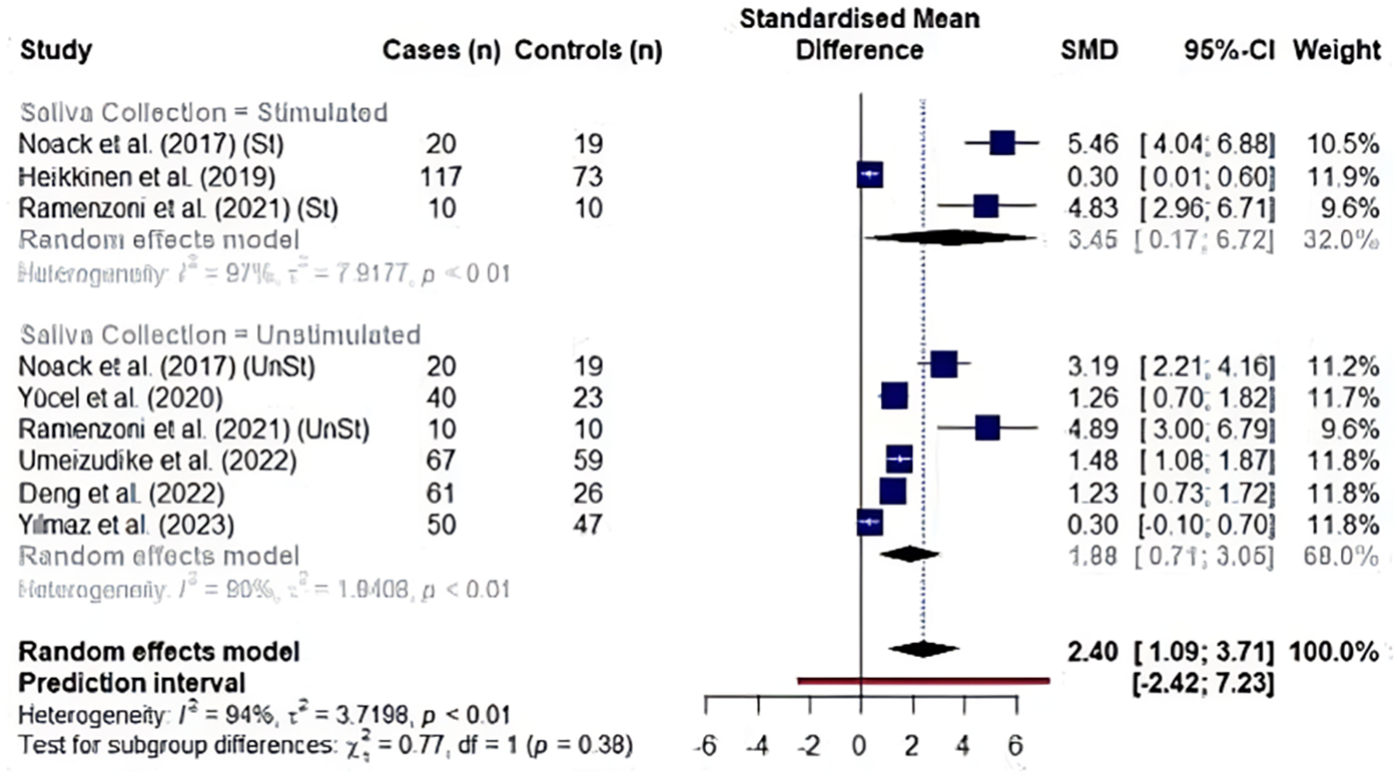
Subgroup analysis by saliva collection technique in studies evaluating aMMP8 levels. St, stimulated saliva collection; UnSt, unstimulated saliva collection. Information on saliva collection method was not reported in studies by Räisänen et al. (2019) (14), Sorsa et al. (2020) (9), Öztürk et al. (2021) (51).

We analyzed the effects of saliva collection techniques on each biomarker. Studies showed an overall effect favoring high MMP8 levels in periodontitis cases (unstimulated saliva collection: SMD: 1.61, 95% CI: 1.04–2.17, *I*^2^ = 92% and stimulated saliva collection: SMD: 0.69, 95% CI: 0.32–1.06, *I*^2^ = 57%). Additionally, saliva collected without prior stimulation had significantly higher MMP8 levels than saliva collected upon stimulation (*p* = 0.007) (Figure 4). In the analysis of aMMP8-quantifying cohorts, both groups showed an overall effect with high aMMP8 levels in periodontitis cases, although the analysis did not improve the heterogeneity (unstimulated: SMD: 1.88, 95% CI: 0.71–3.05, *I*^2^ = 90% and stimulated: SMD: 3.45, 95% CI: 0.17–6.72, *I*^2^ = 97%). With six and three studies in unstimulated and stimulated subgroups, respectively, group results did not differ statistically (*p* = 0.379) (Figure 5).

We also conducted a meta-regression analysis adjusting for the effects of publication year. Analysis with all eligible studies (*I*^2^ = 99.8%, *R*^2^ = 0.0%, *p* = 0.529) and after exclusion of an outlier study by Golitsyna et al. (54) (*I*^2^ = 95.8%, *R*^2^ = 6.9%, *p* = 0.091), showed that publication year was not an affecting predictor of heterogeneity in the studies analyzing MMP8 as a biomarker (Supplementary Figure S1). The same findings were also found in aMMP8-measuring cohorts (*I*^2^ = 97.4%, *R*^2^ = 3.83%, *p* = 0.244) (Supplementary Figure S2).

**Figure 6:**
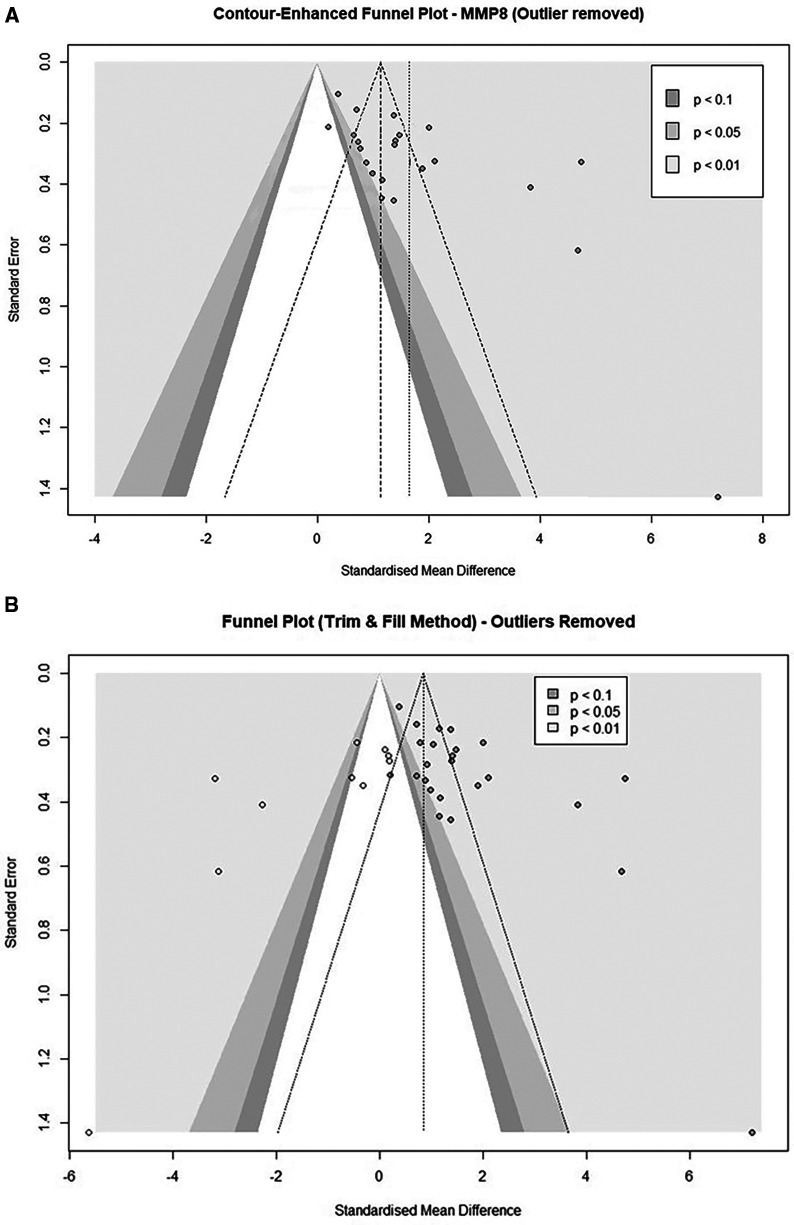
**(A)** Contour-enhanced funnel plot of MMP8-quantifiying studies evaluating the small-study effects after exclusion of an outlier study (*n* = 24). **(B)** Contour-enhanced Funnel plot after a trim and fill analysis (*n* = 24). (Imputed studies are shown with empty dots).

Lastly, we evaluated eligible studies for small-study effects and publication bias. Visualization with a contour-enhanced Funnel plot identified five studies of small-study effects in the MMP8 cohort (48, 54, 56, 58, 65) (Figure 6A and Supplementary Figure S3). Further, Egger's asymmetry test confirmed the presence of funnel plot asymmetry in the cohort of 25 studies (Intercept = 6.464, 95% CI: 3.66–9.26, *t* = 4.52, *p* < 0.001) and the cohort after an outlier study removal (Intercept: 5.161, 95% CI: 2.34–7.98, *t* = 3.587, *p* = 0.002). The trim-and-fill procedure added a total of ten studies and provided an estimate of the corrected effect of 0.87 (95%CI: −2.34 to 4.07, *p* = 0.597) with a heterogeneity of 96.6%. When we applied the adjustment to the study cohorts after an outlier removal (*n* = 24), after the addition of 10 studies, the corrected effect was 0.84 (95% CI: 0.17–1.51, *p* = 0.014) with a heterogeneity of 95.4% (Figure 6B and Supplementary Figure S4).

**Figure 7:**
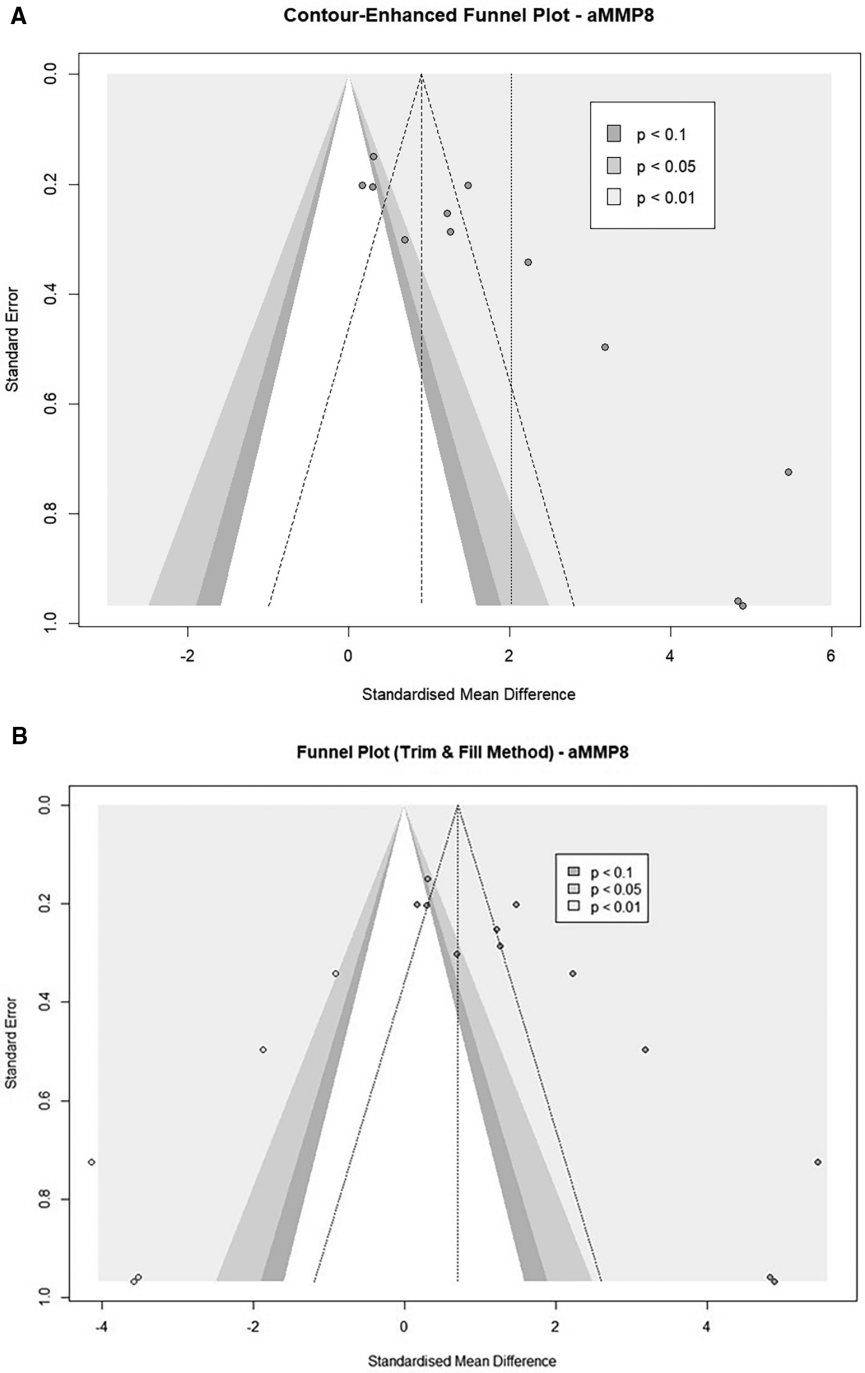
**(A)** Contour-enhanced funnel plots of aMMP8 cohorts (*n* = 12). **(B)** Contour-enhanced Funnel plot after a trim and fill analysis. (Imputed studies are shown with empty dots (*n* = 12).

Funnel plot asymmetry was also observed in five cohorts from three studies using aMMP8 as a biomarker (66, 69, 70) (Figure 7A). An Egger's asymmetry test for small study effects confirmed the funnel plot asymmetry in the general study cohort (Intercept = 6.955, 95% CI: 4.44–9.47, t = 5.427, *p* < 0.001) and after removal of outlier studies (Intercept = 5.694, 95% CI: 3.09–8.3, *t* = 4.284, *p* = 0.004, Supplementary Figure S5). The trim-and-fill-procedure analysis added five studies and provided an estimate of the corrected effect of 0.71 (95%CI: −0.62 to 2.04, *p* = 0.296) with a heterogeneity of 94.6% (Figure 7B). When applied to nine cohorts after the removal of outliers, after adding three studies, the trim-and-fill analysis found a corrected effect of 1.56 (95%CI: 0.08–3.06, *p* = 0.039) with heterogeneity of 91.9% (Supplementary Figure S6).

## Discussion

MMP-8 plays a central role in extracellular matrix remodeling and turnover, contributing to tissue destruction and inflammation with periodontitis events. A total of 25 and 10 studies, respectively, examining MMP-8 and aMMP-8 levels in periodontitis patients compared to healthy controls were included in the current meta-analysis. Our analysis identified significant differences in MMP-8 and aMMP-8 levels between periodontitis cases and healthy controls, supporting their potential as diagnostic biomarkers. Additionally, both biomarkers were similar in overall effect sizes and did not differ statistically.

MMP8 is a widely investigated biomarker in saliva, gingival cervical fluid, and serum. aMMP8 is another biomarker used to quantify metalloproteinase. However, aMMP8, as a biomarker, was introduced to practice later, and the most commonly applied method is point-of-care testing. The limitation in the analysis of aMMP-8 investigating studies was the absence of quantified aMMP8 levels, as the salivary testing reports the presence or absence of the condition defined by a threshold specific to the manufacturers. Also, due to its non-invasive collection method and greater accessibility, potentially enhancing study inclusivity and reducing participant burden, we narrowed our focus to studies using saliva samples to maintain consistency across studies and minimize confounding variables, ensuring more reliable and comparable results in the pooled analysis.

The widespread adoption of MMP8 in both clinical and research settings has established it as a predominant biomarker in numerous studies. Although some research suggests that active MMP-8 (aMMP-8) may more effectively distinguish between healthy sites and those affected by periodontitis, MMP-8 has been found to differentiate between mild and severe periodontitis more accurately in certain cases (10, 74–76). In our study, the sample size for MMP8 was notably larger, involving over 2,000 participants, compared to the aMMP-8 cohorts, which included fewer than 1,000 participants. Additionally, MMP8 studies spanned a broader range of publication years compared to those measuring aMMP8. Despite the potential for publication year to influence heterogeneity due to evolving knowledge, our analysis did not identify publication year as a contributing factor to heterogeneity in effect size.

Initially, MMP8 showed a higher overall effect than aMMP8, though they were statistically similar. However, further analysis revealed that this result was inflated due to the large effect of several studies (46, 47, 54–56). Interestingly, while the removal of indicated studies from both biomarker cohorts slightly increased the effect size of aMMP8 in periodontitis, a drastic and opposite effect was observed in MMP8 studies. The drop in the MMP8 effect was affected mainly by a single study (54); even so, the overall effect of MMP8 became slightly lower than that of aMMP8. However, it should be noted that even without outlier studies, both biomarkers did not differ statistically. Nonetheless, the literature underscores the imperative for further exploration with larger sample sizes to elucidate potential divergent advantages between salivary evaluations of aMMP-8 compared to MMP-8. Our analysis corroborated the observation that both MMP-8 and aMMP-8 levels are elevated in the presence of periodontitis, albeit encountering notably high heterogeneity for both biomarker types.

Our analysis revealed challenges posed by heterogeneity and methodological limitations, prompting an exploration of subgroups not previously evaluated in systematic studies of MMP-8. One area of investigation focused on the type of saliva collection technique used, which yielded variations in MMP-8 levels, with unstimulated saliva exhibiting significantly higher levels compared to stimulated saliva. Numerous epidemiological investigations have frequently regarded unstimulated saliva as a reliable proxy for capturing the holistic microbial composition of the oral environment (77–81). Furthermore, variation in research methodologies, particularly in the composition of biomarkers and microbiota, is evident when comparing stimulated and unstimulated saliva collection methods. While a recent analysis found no difference between the two methods (82), others reported distinct bacterial profiles between stimulated and unstimulated saliva samples, with a notable increase in bacterial diversity observed in stimulated samples, potentially due to the removal of bacterial biofilms during chewing (14, 83–85). Despite the higher MMP-8 levels in unstimulated saliva, we could not indicate the saliva collection technique as a factor leading to heterogeneity between study results.

In our study, we categorized testing into two subgroups: laboratory-based and point-of-care (POC). While we aimed to thoroughly evaluate both, the quantity of POC studies was limited, and these studies often lacked the quantified biomarker levels necessary for our analysis (16, 17, 86, 87). Despite these limitations, prior research highlights the potential advantages of POC testing (88–92). It is not only faster but may also yield more accurate results, likely due to minimized salivary degradation (93, 94). Salivary degradation involves the breakdown of saliva's biomolecules, such as proteins and enzymes, which can occur naturally or be influenced by factors like disease, oral hygiene, or medication (93). This degradation can alter saliva's diagnostic utility, which is why some manufacturers add preservatives to maintain sample integrity until testing. However, the effectiveness of these preservatives, particularly for biomarkers like MMP-8 and aMMP-8, remains underexplored, as most studies focus primarily on preserving DNA or RNA (95–97).

The need for more research into POC salivary diagnostics and biomarker stability is underscored by findings from a meta-analysis conducted at Semmelweis University (80). This study revealed significant heterogeneity in MMP-8 research and noted that different measurement methods, such as enzyme-linked immunosorbent assay (ELISA) and time-resolved immunofluorometric assay (IFMA), reported varied interval levels of MMP-8, with ELISA showing the most significant disparities (MD = 318.12 n, CI: 205.48; 431.37). Similarly, LUMINEX technology, which can analyze up to 100 analytes simultaneously, also demonstrated significant variation (MD = 183.38, CI: 78.92; 187.84) (92). These findings highlight the critical need for further exploration of POC technologies and the factors affecting the stability of salivary biomarkers to enhance their diagnostic accuracy and utility.

Our meta-analysis makes a significant contribution to the literature by synthesizing the available evidence on salivary MMP-8/aMMP-8 levels during periodontitis events. However, notable limitations, such as the observed high heterogeneity across studies and the potential for bias due to publication bias and small sample sizes, must be acknowledged. Despite efforts to decrease heterogeneity by separating the studies into subgroups based on type of saliva collection method, year of publication, and different study populations, the heterogeneity remained considerable. The impact of study design on heterogeneity and effect estimates cannot be overlooked. Variations in inclusion criteria, sample characteristics, saliva collection methods, and MMP-8/aMMP-8 measurement techniques across studies may contribute to heterogeneity in our meta-analysis (98). While subgroup analyses were performed to explore sources of heterogeneity, residual variability may still exist due to unmeasured confounders or methodological differences not accounted for in our study.

It is crucial to consider the potential for publication bias, where studies with statistically significant results are more likely to be published, leading to an overestimation of effect sizes (99). While efforts were made to include all relevant studies through a comprehensive literature search, the possibility of unpublished or inaccessible data cannot be entirely ruled out. This bias could have influenced our meta-analysis results, particularly in studies reporting small effect sizes or non-significant findings. In addition, selective outcome reporting bias may exist, whereby studies selectively report outcomes based on their statistical significance, leading to an overrepresentation of positive results. To mitigate this bias, we meticulously screened studies for inclusion based on predefined criteria and conducted various analyses to assess the robustness of our findings. However, the potential for selective outcome reporting bias should be considered when interpreting our results.

In reviewing the existing data and additional subgroup analysis, it becomes evident that the observed high heterogeneity within our findings highlights the necessity for standardized protocols. These protocols are essential for ensuring the reliability and reproducibility of results when evaluating salivary biomarkers. Further, it emphasizes the need for consistent calibration of reference intervals across different saliva collection methods, testing devices, and specific biomarkers being assessed. Standardization is particularly vital to ensure accuracy in the diagnosis and monitoring of periodontitis using saliva-based testing. Such standardization typically involves studies that establish biomarker interval ranges in saliva correlated with clinical assessments of disease severity. The Clinical and Laboratory Standards Institute (CLSI) offers guidelines for defining, establishing, and verifying reference intervals, which include setting the upper and lower reference limits that are designed to encompass a specific percentage (95%) of the population values from selected reference subjects (100). For most analytes, these limits are calculated as the 2.5th and 97.5th percentiles of the distribution of test results in the reference population. Future research should focus on standardizing saliva collection protocols to reduce variability and improve reproducibility, while also exploring MMP-8's diagnostic relevance across diverse populations, including variations by age, ethnicity, and systemic health conditions.

Our study also underscores the importance of future research to mitigate these issues by implementing standardized methodologies and expanding sample sizes. This approach is crucial to improve diagnostic precision and the clinical application of saliva-based tests in evaluating periodontitis. Additionally, it is important to consider diverse patient populations characterized by various factors such as smoking habits, diabetes status, and dietary practices, including snacking frequency, all of which may influence MMP-8 levels (101–104). Conducting studies in real-world settings with larger, diverse cohorts and standardized methods will help address current limitations and provide strong evidence that supports the integration of saliva-based diagnostics into standard dental care practices. This advancement will significantly enhance the understanding and management of periodontitis.

## Conclusion

The current meta-analysis provides compelling evidence of the association between salivary MMP-8/aMMP-8 levels and periodontitis. These findings underscore the utility of saliva as a non-invasive diagnostic tool and highlight the intricate role of MMP-8 in the pathophysiology of periodontitis.

Our results highlight the promising role of saliva-based diagnostics in enhancing the management of periodontitis. Despite encountering challenges such as heterogeneity and methodological limitations, the accessibility and convenience of saliva collection make it a valuable tool for routine screening and monitoring. Collaborative efforts, standardized protocols, and larger studies are needed to validate salivary biomarkers and improve oral health.

## Data Availability

The original contributions presented in the study are included in the article/Supplementary Material, further inquiries can be directed to the corresponding author.

## Supplementary material

Supplementary Figure S1 Bubble plot of meta-regression analysis evaluating MMP8 values adjusted for the publication year illustrating the estimated regression slope and effect size of included studies. [The outlier study by Golitsyna et al. (36) has been excluded].

Supplementary Figure S2 Bubble plot of meta-regression analysis evaluating aMMP8 values adjusted for the publication year illustrating the estimated regression slope and effect size of included studies.

Supplementary Figure S3 Funnel plot of MMP8-quantifying studies with all eligible studies (*n* = 25).

Supplementary Figure S4 Funnel plot after a trim and fill analysis. (Imputed studies are shown with empty dots) (*n* = 25).

Supplementary Figure S5 Funnel plot of aMMP8 studies after exclusion of outlier cohorts (*n* = 9).

Supplementary Figure S6 Contour-enhanced Funnel plot after a trim and fill analysis. (Imputed studies are shown with empty dots) (*n* = 9).

## Appendix

**Appendix 1:** Search Strategy using MeSH and TIAB.

Aspect	MeSH + TIAB	Comments
MMP-8	"Matrix Metalloproteinase 8"[Mesh] OR “Matrix Metalloproteinase 8"[tiab] OR “MMP 8”[tiab] OR “MMP8”[tiab] OR “MMP-8"[tiab] OR "Neutrophil Collagenase"[tiab] OR “PMN Collagenase"[tiab] OR “Collagenase 2"[tiab] OR “CLG1"[tiab] OR	"EC 3.4.24.34"[tiab]
Diagnosis accuracy/prediction	"Diagnosis"[Mesh] OR “Biomarkers"[Mesh] OR “diagnosis"[Subheading] OR “diagnos*”[tiab] OR “Early Detect*”[tiab] OR Predict*[tiab] OR “Identif*”[tiab] OR “Recognition”[tiab] OR “Screening”[tiab] OR “Confirmatory Test*”[tiab] OR “Clinical Assessment*”[tiab] OR biomarker*[tiab] OR marker*[tiab] OR indicator*[tiab]	"Early Diagnosis"[Mesh] OR
Gingivitis OR periodontal inflammation	"Gingivitis"[Mesh] OR “Gingivitis, Necrotizing Ulcerative"[Mesh] OR Gingiviti*[tiab] OR “Gingiva inflam*”[tiab] OR “Gingival inflam*”[tiab] OR “Gingival Infection”[tiab] OR “Gingival Swelling”[tiab] OR Gingival Erythema”[tiab] OR “Gum Dis*”[tiab] OR “Gum Infection”[tiab] OR “Gum Swelling”[tiab] OR “Gum Redness”[tiab] OR “Gum Inflam*”[tiab] OR “Inflammation of gum tissue” [tiab] OR “Inflamed Gum*”[tiab] OR “Swollen Gum*”[tiab] OR “Inflammation of the Periodontium” [tiab] OR “Periodontitis”[tiab] OR “Periodontal Dis*”[tiab] OR “Periodontal Tissue Dis*”[tiab] OR “Periodontal Tissue Infect*”[tiab] OR “Periodontal Tissue Inflam*”[tiab] OR “Periodontitis”[tiab] OR “Periodontal Swelling”[tiab] OR “Periodontal Inflam*”[tiab] OR “Periodontal Infect*”[tiab] OR “Periodontal Erythema”[tiab] OR	
