# Supplementary figures and images for: Assessment of salivary matrix metalloproteinase (MMP8) and activated salivary matrix metalloproteinase (aMMP8) in periodontitis patients: a systematic review and meta-analysis
Source: Front Oral Health. 2025 Feb 19;6:1444399. doi: 10.3389/froh.2025.1444399 (PMC11880025; doi:10.3389/froh.2025.1444399)

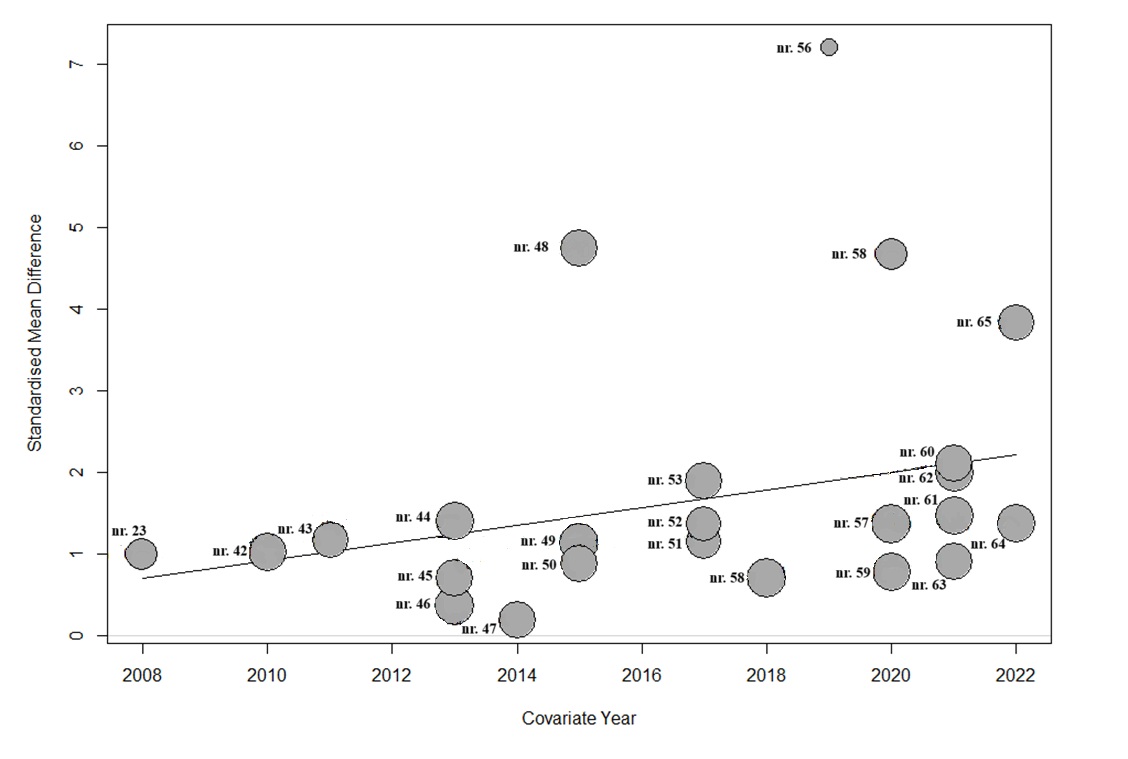

Supplement: Supplementary Figure S1 — Bubble plot of meta-regression analysis evaluating MMP8 values adjusted for the publication year illustrating the estimated regression slope and effect size of included studies. [The outlier study by Golitsyna et al. (36) has been excluded]. [file Image1.jpeg]

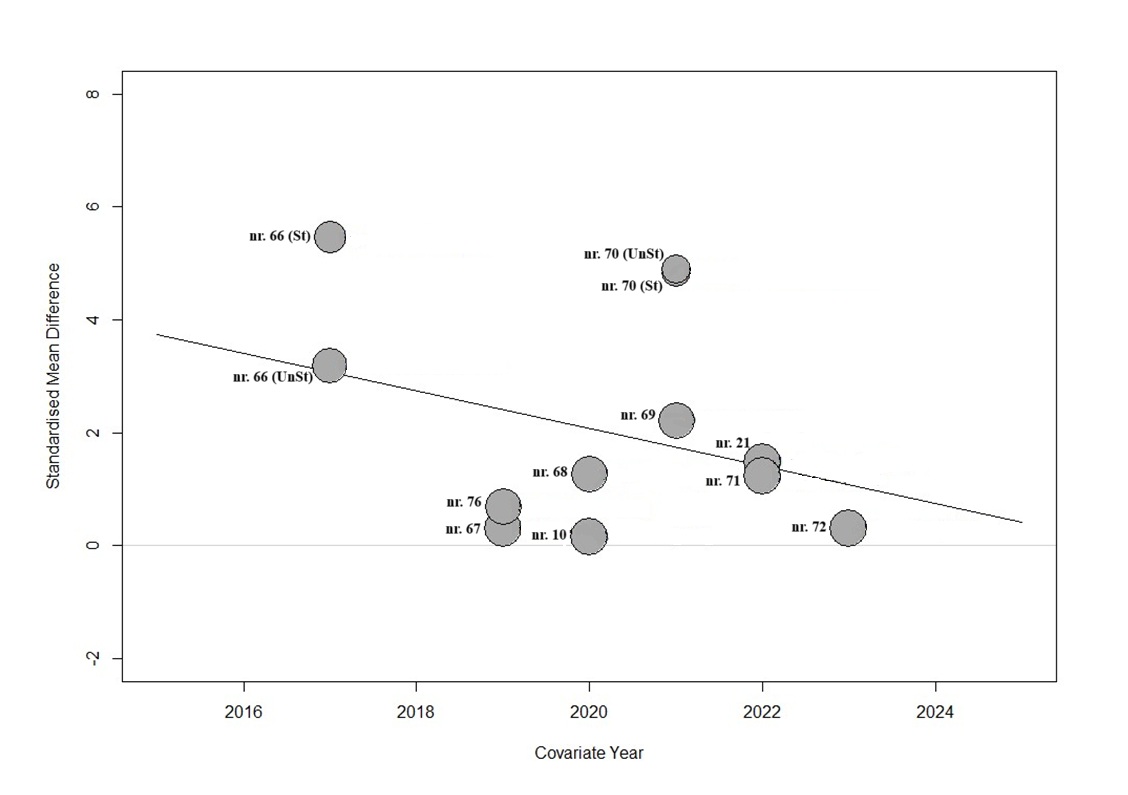

Supplement: Supplementary Figure S2 — Bubble plot of meta-regression analysis evaluating aMMP8 values adjusted for the publication year illustrating the estimated regression slope and effect size of included studies. [file Image2.jpeg]

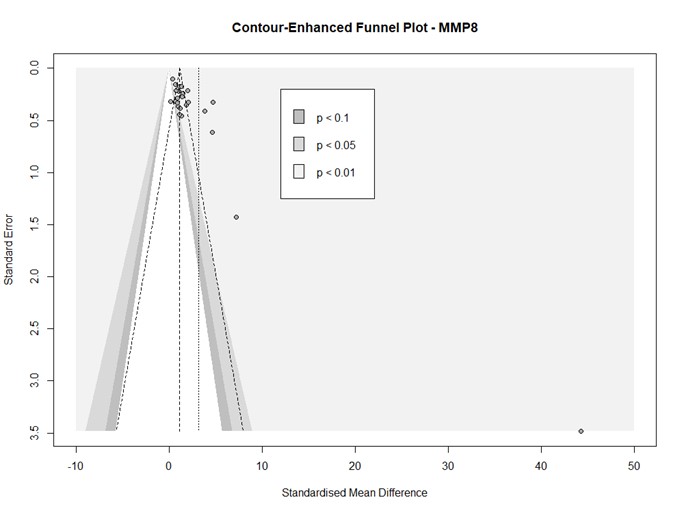

Supplement: Supplementary Figure S3 — Funnel plot of MMP8-quantifying studies with all eligible studies (n = 25). [file Image3.jpeg]

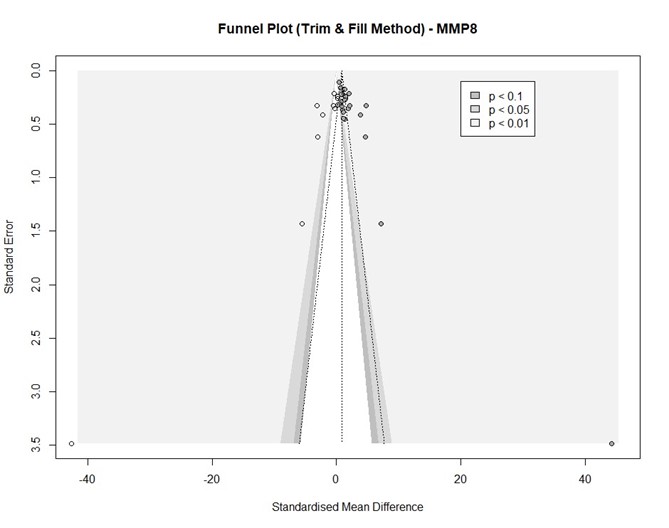

Supplement: Supplementary Figure S4 — Funnel plot after a trim and fill analysis. (Imputed studies are shown with empty dots) (n = 25). [file Image4.jpeg]

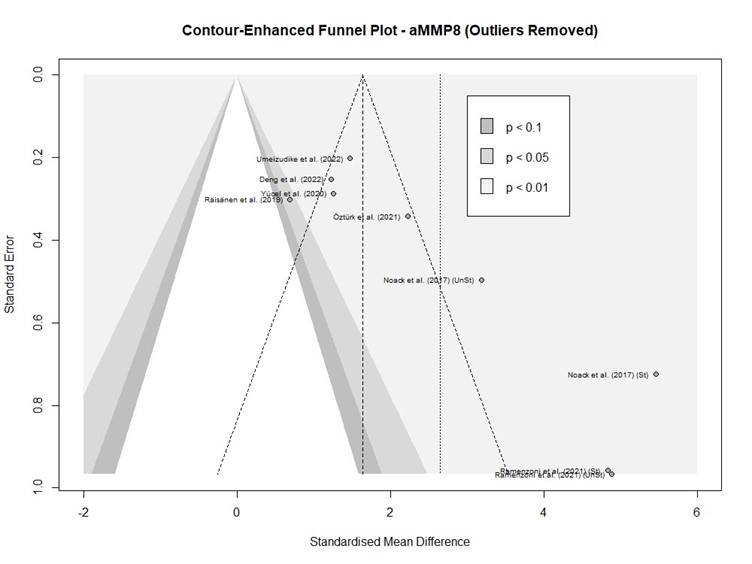

Supplement: Supplementary Figure S5 — Funnel plot of aMMP8 studies after exclusion of outlier cohorts (n = 9). [file Image5.jpeg]

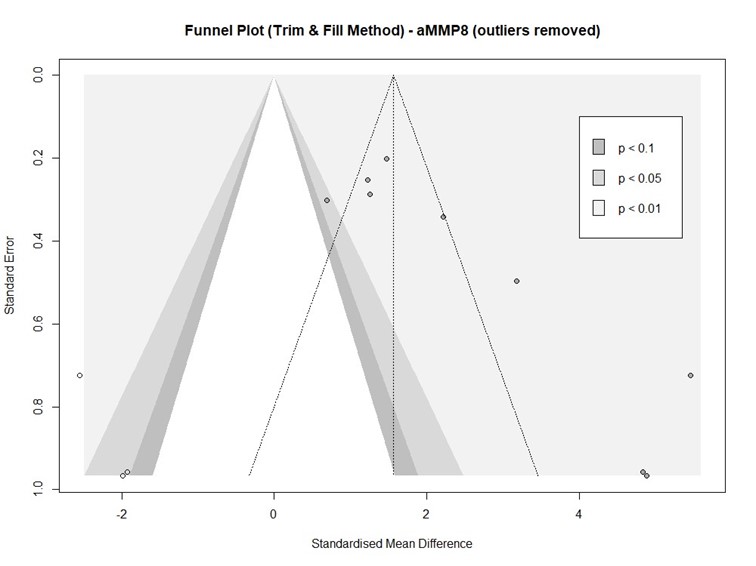

Supplement: Supplementary Figure S6 — Contour-enhanced Funnel plot after a trim and fill analysis. (Imputed studies are shown with empty dots) (n = 9). [file Image6.jpeg]
